# Glycopeptide-Based Supramolecular Hydrogels Induce Differentiation of Adipose Stem Cells into Neural Lineages

**DOI:** 10.1021/acsami.3c05309

**Published:** 2023-06-16

**Authors:** Vânia I.B. Castro, Ana R. Araújo, Filipa Duarte, António Sousa-Franco, Rui L. Reis, Iva Pashkuleva, Ricardo A. Pires

**Affiliations:** † 3B’s Research Group, I3Bs—Research Institute on Biomaterials, Biodegradables and Biomimetics, University of Minho, Headquarters of the European Institute of Excellence on Tissue Engineering and Regenerative Medicine, 4805-017 Guimarães, Portugal; ‡ ICVS/3B’s—PT Government Associated Laboratory, 4805-017 Braga/Guimarães, Portugal

**Keywords:** glycopeptide, supramolecular, hydrogels, stem cell differentiation, neural tissue

## Abstract

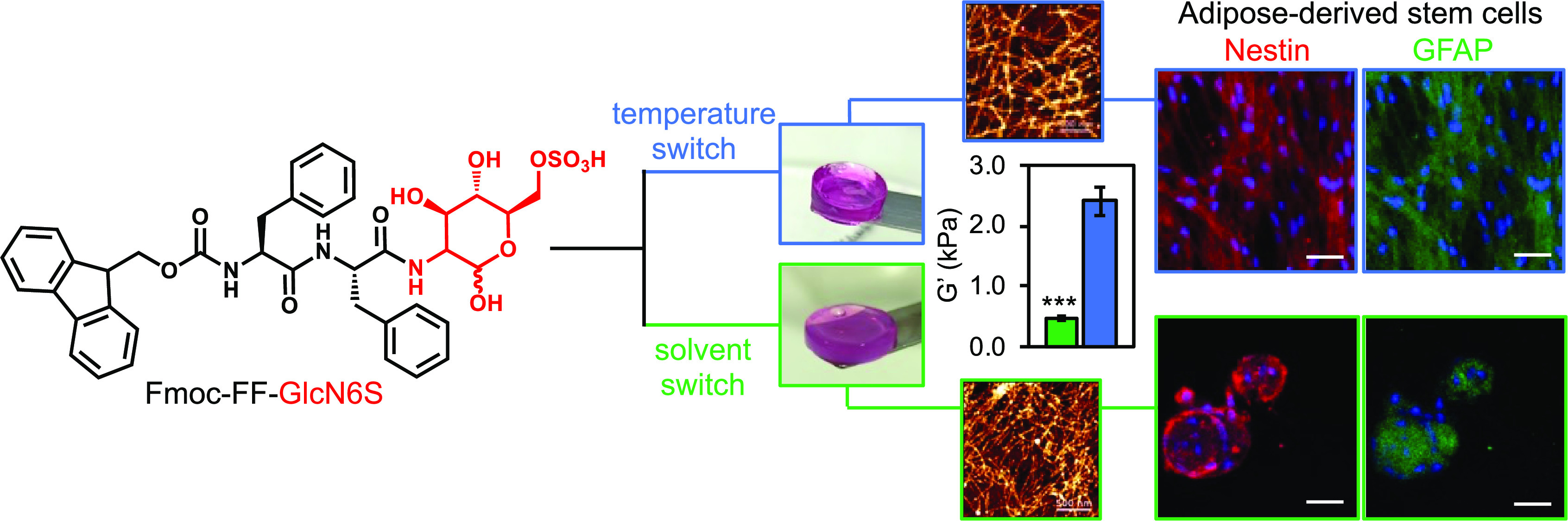

We applied a bottom-up approach to develop biofunctional supramolecular hydrogels from an aromatic glycodipeptide. The self-assembly of the glycopeptide was induced by either temperature manipulation (heating–cooling cycle) or solvent (DMSO to water) switch. The sol–gel transition was salt-triggered in cell culture media and resulted in gels with the same chemical compositions but different mechanical properties. Human adipose derived stem cells (hASCs) cultured on these gels under basal conditions (i.e., without differentiation factors) overexpressed neural markers, such as GFAP, Nestin, MAP2, and βIII-tubulin, confirming the differentiation into neural lineages. The mechanical properties of the gels influenced the number and distribution of the adhered cells. A comparison with gels obtained from the nonglycosylated peptide showed that glycosylation is crucial for the biofunctionality of the hydrogels by capturing and preserving essential growth factors, e.g., FGF-2.

## 1 Introduction

The extracellular matrix (ECM) is a complex and dynamic entity, which serves as a support for cell attachment and provides biochemical and mechanical cues determining cell fate.^1,2^ It is composed mainly by proteins and proteoglycans that are organized in a fibrous hydrated mesh.^1−3^ Supramolecular gels, generated by unidirectional self-assembly of peptides, have been used to create biofunctional ECM mimics.^3−7^ These gels share structural similarities with the ECM: they are highly hydrated nanofibrous scaffolds assembled by multivalent noncovalent interactions.^3,4,8^ The design of self-assembling peptides as building blocks for such gels has been inspired by the ability of proteins to self-organize into three-dimensional structures through the formation of intermolecular hydrogen bonds.^5,6^ In the case of short peptides, the hydrogen bonding often need to be complemented by other attractive interactions (e.g., electrostatic, hydrophobic, or aromatic stacking) to achieve the formation of stable fibers. Thus, a functionalization of the peptide with charged, hydrophobic, or aromatic functionality is often applied.^9−12^

The biofunctionality of such supramolecular ECM mimics is set primarily by the amino acid sequence that usually copycats proteins with known bioactivity. In the native ECM, however, glycans and proteoglycans also code bioinformation.^13,14^ One example is heparan sulfate (HS) composed by alternating sulfated *N*-acetylglucosamine (GlcNAc) and glucuronic acid (GlcA). HS-proteoglycans are abundant in the ECM of the central nervous system (CNS) where they are involved in neurogenesis, axon guidance, and synaptogenesis.^14,15^ In these processes, the number of HS chains attached to the protein core and their sulfation pattern are crucial as they define binding sites for growth factors and other molecules.^14,15^ Moreover, the carbohydrate moieties influence the ECM viscoelastic properties by enhancing the hydration and hydrophilicity and altering the conformational stability of the core proteins.^16−18^

The important involvement of glycans in cell-ECM signaling motivated the development of supramolecular hydrogels based on biofunctional glycopeptides.^19−24^ The design of glycopeptide building blocks for ECM mimicking gels is challenging because the introduced carbohydrate functionality can affect the hydrophobic balance and the stereochemistry of the block, as we have previously shown for minimalistic tripeptides, FSF and FTF, conjugated with glucose at serine (S) or tyrosine (T)^25^ and as others have shown for different glycosylated pentapeptides.^26^ As a result, the glycosylation can perturb the supramolecular interactions driving the assembly of the respective nonglycosylated peptide blocks.^25−27^ The synthesis is also demanding as multiple reactive groups are available at both the carbohydrate and peptide moieties and need to be protected selectively to obtain a targeted glycoconjugate. Alternatively, co-assembly of a peptide gelator with glycan amphiphiles has been proposed as a simpler modular approach.^28,29^ In this case, however, the control over the spatial distribution of the carbohydrate along the peptide-based nanofiber is challenging.

**Figure 1:**
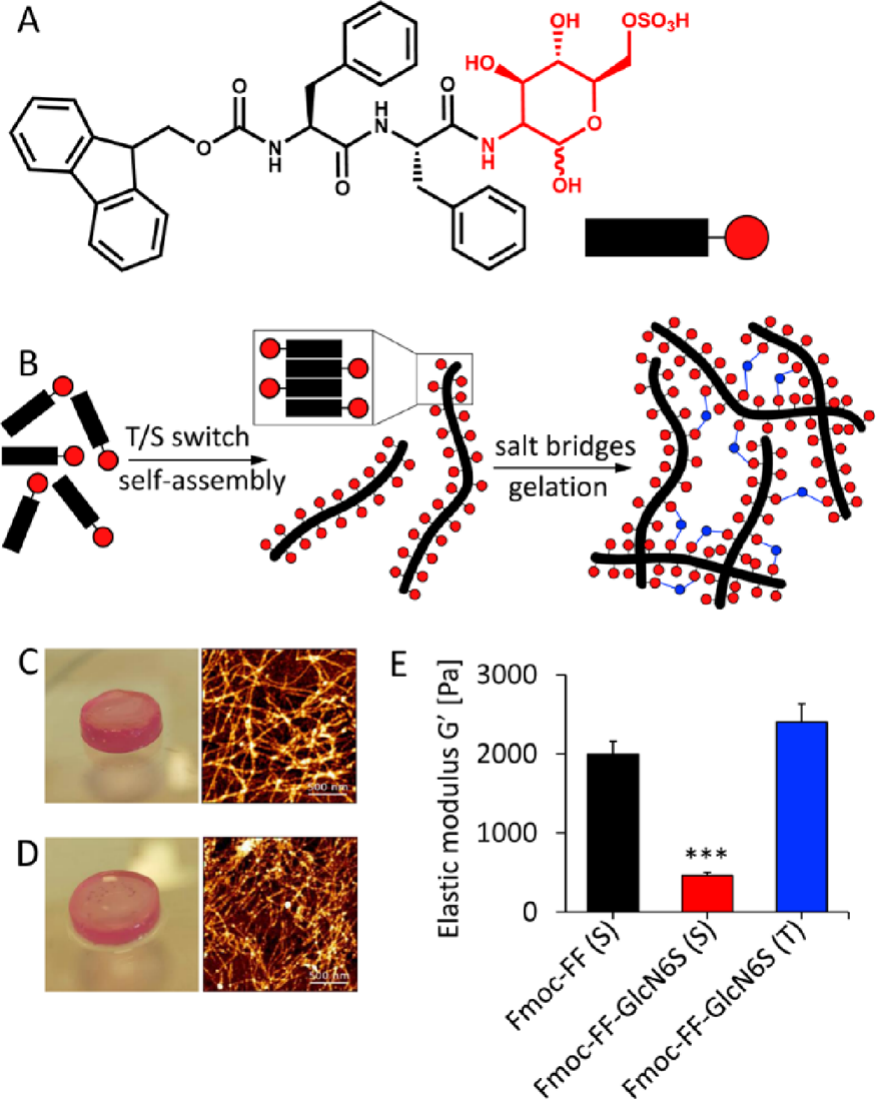
(A) Chemical structure of the glycopeptide Fmoc-FF-GlcN6S and (B) schematic presentation of its self-assembly triggered by temperature (T) or solvent (S) switch and following gelation in the presence of divalent cations. (C, D) Macroscopic appearance of the gels obtained from the glycopeptide (10 mM, culture medium) by the (C) T and (D) S method and their nanostructure imaged by atomic force microscopy (AFM, scale bar 500 nm). (E) Elastic modulus (*G*′, 37 °C) of the gels prepared from the glycopeptide and its nonglycosylated analogue Fmoc-FF (S method). Statistics: *** *p* < 0.001.

Herein, we propose a simple glycopeptide gelator (Figure 1A) prepared by coupling of Fmoc-diphenylalanine (Fmoc-FF) and glucosamine-6-sulfate (GlcN6S) using standard carbodiimide chemistry (Figures S1–S5). We demonstrate that different methodologies can be applied to obtain gels from this glycopeptide and that glycosylation has an impact on the conformation of the assemblies. Importantly, the conjugated GlcN6S strikingly enhances the biocompatibility and biofunctionality of the generated gels.

## 2 Materials and Methods

### 2.1 Materials

Solvents and reagents were used without further purification. Fmoc-Phe-Phe-OH (Fmoc-FF, purity 98.95%) was purchased from Bachem, Switzerland. d-Glucosamine-6-*O*-sulfate (GlcN6S, purity 99.0%) was acquired from Carbosynth, U.K. Coupling reagents (*N*,*N*′-dicyclohexylcarbodiimide (DCC) and *N*-hydroxysuccinimide (NHS)) and solvents (anhydrous tetrahydrofuran (THF)) were acquired from Sigma, Portugal.

### 2.2 Synthesis, Purification, and Characterization of the Glycopeptide Fmoc-FF-GlcN6S

The glycopeptide was obtained following a standard carbodiimide protocol (Figure S1). Briefly, Fmoc-FF (1 mmol) was dissolved in dry THF (20 mL). DCC (1.2 equiv) and NHS (1.2 equiv) were added to the solution of the peptide, and the reaction mixture was stirred for 4 h at 4 °C under a nitrogen atmosphere. Afterward, the mixture was filtered to remove any solid precipitate, and the carbohydrate GlcN6S (2 equiv) and NaHCO_3_ (6 equiv) dissolved in water (4 mL) were added dropwise to the filtered solution. The reaction was stirred for 18 h at 4 °C. The solvents were removed (rotary evaporation under a vacuum), and the obtained solid was washed with ethyl acetate (AcOEt).

After freeze-drying, the glycopeptide was purified by preparative high-performance liquid chromatography (HPLC) using a Waters 2545 Binary Gradient Module HPLC system with an Atlantis preparative T3 C18 Column (5 μm, 30 × 250 mm). A gradient of water (A) and acetonitrile (B), a flow of 12 mL/min, and a loop of 5 mL were used for the purification. The gradient was set as follows: an initial mixture of 80% A and 20% B was gradually changed to 45% A and 55% B (over 15 min) followed by a further increase of B until 100% at 25 min. This gradient was maintained during 5 min and then changed (over 5 min) to a gradient of 80% A and 20% B that was maintained for an additional 5 min. The glycopeptide was characterized by mass spectroscopy (Quattro Micro API, Waters Corporation, U.K.) in negative-ion mode (Figure S2), nuclear magnetic resonance (NMR, 400.13 MHz Avance III spectrometer, Bruker, Germany) in DMSO-*d*_6_ (Figure S3), and Fourier transform infrared spectroscopy (FTIR, KBr pellets, IR-Prestige 21 spectrometer, Shimadzu, Japan, Figure S4). The purity was determined by an analytical HPLC (Smart Line, Knauer) with reverse-phase C18 Atlantis column (5 μm, 250 × 4.6 mm, Waters, U.K.). We used a gradient of water (A) and acetonitrile (B) with 0.1% of TFA as buffer, a flow of 1 mL/min, and a loop of 50 μL. In the beginning, the eluent was composed by 80% A and 20% B that were gradually changed to 20% A and 80% B (over 35 min) and then to 100% B (during 3 min). This eluent was maintained for 5 min, gradually changed to a gradient of 80% A and 20% B (over 5 min), and an isocratic elution with this eluent for 5 min. For all the experiments, we used a glycopeptide sample with purity >96% (Figure S2).

### 2.3 Self-Assembly and Gelation

#### 2.3.1 Self-Assembly Induced by Temperature Switch (T Method)

Fmoc-FF-GlcN6S (e.g., 10 mM; 7.6 mg/mL) was suspended in water. The temperature was increased to 90 °C during 2–3 min until complete dissolution of the glycopeptide (formation of a clear solution) and then decreased to induce self-assembly and formation of a viscous solution (pregelation solution). Pregelation solutions with different concentrations (5 and 7.5 mM) were also prepared for comparative purposes.

#### 2.3.2 Self-Assembly Induced by Solvent Switch (S Method)

Fmoc-FF-GlcN6S or Fmoc-FF was dissolved in DMSO at a concentration of 0.2 M (7.6 mg/50 μL for Fmoc-FF-GlcN6S and 10.0 mg/50 μL for Fmoc-FF) to obtain a stock solution. The self-assembly was induced by the addition of water (volume needed to achieve the intended final concentration of 10 mM) to the stock solution. At these conditions, we obtained transparent viscous solutions (pregelation solution).

#### 2.3.3 Gelation

Pregelation solutions were prepared using the S or T method as described above. Three hundred microliters of the pregelation solution (e.g., 10 mM) was added into a 12-well Thincert cell culture insert (Greiner Bio-One, GmbH, Kremsmünster, Austria). The inserts loaded with the pregelation solution were placed into 12-well culture plates, and the medium (1.5 mL) was added to each well. The plate was incubated for 3 h at 37 °C and 5% CO_2_. Afterward, the medium was replaced with a new one. At this stage, the medium (250 μL) was also added to the top of the inserts to cover the gels. During the following 24 h of incubation, the medium (top of the insert and bottom of the well) was replaced one more time. After this period, the gels were characterized.

### 2.4 Characterization of the Gels

#### 2.4.1 Circular Dichroism (CD) Spectroscopy

Circular dichroism (CD) spectra were recorded in a Jasco J1500 spectrophotometer (Jasco Corporation, Japan) using a quartz cuvette with 0.1 mm path length. Spectra of the different pregelation solutions and hydrogels (at a concentration of 10 mM) were recorded at a scan speed of 200 nm.min^–1^ with a data pitch of 0.5 nm and a bandwidth of 2.0 nm. For all conditions, a triple acquisition was performed, and the solvent background was subtracted from the obtained spectra.

#### 2.4.2 Fluorescence Emission Spectroscopy

A Jasco FP-8500 spectrofluorometer (Jasco Corporation, Japan) was used to record the fluorescence emission spectra of the pregelation solution. Pregelation solutions (10 mM) were deposited in a black 96-well plate, and the fluorescence was measured using a microplate accessory. An excitation at 265 nm was used, and the emission spectra were recorded in the range 300–600 nm. All readings were performed using a bandwidth of 5 nm with a 50 ms response and a 1 nm data pitch.

#### 2.4.3 Attenuated Total Reflectance Fourier Transform Infrared (ATR-FTIR) Spectroscopy

The ATR-FTIR spectra were recorded on an IR Prestige-21 spectrometer with an ATR module (Shimadzu Corporation, Japan). Gels (10 mM) were deposited on a glass slide and vacuum dried. Spectra of the dried gel samples were recorded at room temperature with a resolution of 4 cm^–1^ over 128 accumulations. The signal from the glass slides (background) was also recorded and subtracted from the obtained spectra. The IR data in the region 1600–1800 cm^–1^ were then fitted by multiple Gaussian peaks, and the proportion of each secondary structure was calculated.

#### 2.4.4 Atomic Force Microscopy (AFM)

Freshly cleaved mica sheets were rinsed with deionized water and dried under a nitrogen flow. For the pregelation samples, a drop of the viscous solution (10 mM) was deposited on the mica sheet. For gel samples, a mica sheet was placed on the top of the gel (10 mM) for 1 min, washed with water, and dried under a gentle stream of nitrogen. AFM images were acquired using JPK Nanowizard 3 (JPK, Germany) in air at room temperature under AC mode. The scans were acquired at a 512 × 512 pixel resolution using ACTA-SS probes (*k* ∼37 N/m, AppNano, Scientec, France), a drive frequency of ∼254 kHz, a set point of ∼0.5 V, and a scanning speed of 1.0 Hz. Images were analyzed using the JPK data processing software. The fiber diameter was determined from the generated AFM images using the ImageJ software.

#### 2.4.5 Oscillatory Rheology

Mechanical properties of the hydrogels were assessed using a strain-controlled rheometer (Kinexus Pro, Malvern, U.K.) equipped with 8 mm parallel-plate geometry. The samples were placed on the plate, and the gap was adjusted. All measurements were performed at 37 °C. First, a linear viscoelastic region (LVR) was determined for each sample, and amplitude sweeps at a frequency of 1 Hz and 0.1–10% strain were applied. Taking into consideration the LVR results, frequency sweeps were performed at 1% strain. Measurements of storage modulus (*G*′) and loss modulus (*G*″) were acquired at a frequency of 1 Hz and a strain of 1%. All measurements were repeated three times to ensure reproducibility, and data are reported as average values.

#### 2.4.6 Ability of the Gels to Self-Heal

To assess the self-healing ability of the gels (10 mM), we evaluated the response of the gels to the applied shear force at 37 °C. We used a rheometer (Kinexus Pro, Malvern, U.K.) equipped with an 8 mm parallel-plate geometry. Gels were prepared 24 h before the measurements. Critical strain values were determined from the point where *G*′ values start to decrease with strain. Shear force was applied following the procedure in terms of strain (%) and duration (s): 0.1% (200 s), 0.1% (200 s), 100%(200 s), 0.1%(200 s), 100(200 s), 0.1%(200 s), 100% (200 s), and 0.1% (200 s).

#### 2.4.7 Degradability of the Hydrogels

Gels (10 mM) were prepared as described above and maintained under standard cell culture conditions, i.e., culture medium, 5% CO_2_, and 37 °C. At predetermined time points, namely, 2, 10, and 21 days, the gels were dissolved in acetonitrile and analyzed by analytical HPLC as described above.

### 2.5 Biological Assessment of the Gels

#### 2.5.1 Assessment of Biocompatibility

Cytotoxicity assays with different cell lines, namely, mouse fibroblast cells (L929), neuroblastoma cell line (SH-SY5Y), osteosarcoma cell line (SaOs-2), chondrogenic cell line (ATDC5), as well as primary human adipose derived stem cells (hASCs), were performed. ATDC5 and hASCs were expanded in α-Minimum Essential Medium Eagle (α-MEM; Gibco, U.K.) supplemented with 10% of fetal bovine serum (FBS, Gibco, U.K.) and 1% antibiotic antimycotic solution (ATB; Gibco, U.K.) and incubated at 37 °C in the presence of 5% CO_2_ until confluence. SH-SY5Y were grown in Dulbecco’s modified Eagle’s medium nutrient mixture F-12 (DMEM-F12; Gibco, U.K.) supplemented with 10% FBS and 1% ATB solution. SaOs-2 cells were grown in Dulbecco’s modified Eagle’s medium low glucose (DMEM-LG; Sigma-Aldrich, U.K.) supplemented with 10% FBS and 1% ATB solution.

Fmoc-FF and Fmoc-FF-GlcN6S were sterilized by UV irradiation for 30 min, and then the gels were prepared at sterile conditions as described in Section 2.3.3. The culture medium from the insert (top of the gel) was replaced with cell suspension (200 μL, 5 × 10^4^ cells/gel) in the respective media. Cellular viability was evaluated by a live/dead assay. Briefly, at predetermined time points, gels with cells were incubated in PBS with calcein AM (diluted 2:1000; Biotium, USA) and ethidium homodimer-1 (EthD-1; diluted 1:1000; Sigma-Aldrich, U.K.) for 30 min at 37 °C. Then, the gels were removed from the insert, placed on microscope glass slides, and observed under a confocal laser scanning microscope (TCS SP8, Leica, Germany).

#### 2.5.2 Assessment of Biocompatibility

Cellular viability was assessed for hASCs using an AlamarBlue Cell Viability Assay (Biorad, U.K.). hASCs were seeded (2 × 10^4^ cells/gel) on the top of the gels (10 mM). After predetermined culture times, the medium was carefully aspirated, and the hydrogels were rinsed with 20% (v/v) AlamarBlue Cell Viability Assay solution in α-MEM for 5 h at 37 °C with 5% CO_2_. Following incubation with the reagent, aliquots (100 μL) were placed into black 96-well plates, and the fluorescence was measured using a plate reader (Synergy, Bio-Tek, USA) with an excitation wavelength of 550 nm and an emission wavelength of 590 nm. Cells cultured on tissue culture polystyrene (TCPS) were used as controls, and acellular gels were used as blank (to adjust for background fluorescence).

#### 2.5.3 Cell Organization and Morphology

Assessment of cellular organization on different gels was performed by contrast image microscopy with an inverted microscope (Primovert, Zeiss, Germany), and cells were imaged without any staining applied. Cell morphology was visualized by staining of the actin cytoskeleton. Briefly, gels with the cultured cells were treated with 0.1% Triton-X 100 at 4 °C for 5 min and then incubated with phalloidin (5:1000 in 1% BSA; Cytoskeleton, USA) for 45 min at 37 °C. A drop of 4′,6-diamidino-2-phenylindole (DAPI (1:1000); ThermoFisher Scientific, Netherlands) was also added 2 min before observation. The stained samples were removed from the inserts, placed on glass microscope slides, and observed under a confocal laser scanning microscope (TCS SP8, Leica, Germany).

#### 2.5.4 Gene Expression Analyses

At predetermined cell culture periods, the medium was removed from the gels and washed with phosphate buffer saline (PBS). Trizol (350 μL/gel) (ThermoFisher Scientific, Netherlands) was then added to the insert with the gel and cells and incubated for 5 min. The gel was destroyed (pipetting up and down); the total volume of Trizol + gel was transferred to an Eppendorf tube, and chloroform (120 μL/gel) was added. The mixture was vortexed and centrifuged (4 °C, 13,000*g*, 20 min), the aqueous phase was collected, and 70% ethanol was added (1:1 v/v). At least three gels per condition were pooled to allow collection of sufficient material for analysis. The samples were run in RNeasy Kit spin columns (Qiagen, USA), and RNA was purified according to the manufacturer’s instructions. The obtained RNA was treated with amplification-grade DNAseI (Merck Life Science, Portugal) according to the manufacturer’s instructions and quantified using a NanoDrop spectrophotometer (1000, ThermoScientific, USA).

Reverse transcription reaction was performed from 250 ng of initial RNA samples using a Quantabio qScript cDNA synthesis kit (Quantabio, USA) according to the manufacturer’s instructions in a MiniOpticon thermocycler (CFD3121, BioRad, USA). Real-time quantitative PCR was performed using a PerfeCTa SYBR Green fastmix reagent (Quantabio, USA) according to the manufacturer’s instructions in a Reverse Transcription Polymerase Chain Reaction (RT-PCR)-Mastercycler (Realplex, Eppendorf, Germany). Sample cDNA obtained from the previous cDNA synthesis step was diluted in RNAse-free water to a final concentration of 2 ng/μL, from which a sample of 5 μL was used for each amplification reaction, in triplicates. Primers used for amplification (Table S1) were designed using Primer3Plus.^30^

#### 2.5.5 Immunocytochemistry Assay

Samples (gels with cells) were washed with PBS and fixed with 4% (w/v) paraformaldehyde (Alfa Aesar, U.K.) at 4 °C overnight. Afterward, the samples were washed again with PBS, and cells were permeabilized with 0.1% Triton-X 100 (4 °C, 5 min) followed by a blocking treatment with 3% bovine serum albumin (BSA) in PBS (5 min at 37 °C). Staining was performed by incubation (1 h at 37 °C) with primary antibodies, namely, human anti-Nestin (diluted 1:200 in 3% BSA, MAB5326 Merck, Germany), goat anti-GFAP (diluted 1:200 in 3% BSA, sc-6171, Santa Cruz, USA), rabbit anti-β3-tubulin (diluted 1:400 in 3% BSA, ab18207, Abcam), and rabbit anti-MAP2 (diluted 1:750 in 3% BSA, 840601, Biolegend, USA). Samples were washed (with PBS) and incubated with an appropriate fluorescent secondary antibody. First, cells were incubated with donkey anti-mouse Alexa Fluor-594 or anti-rabbit Alexa Fuor-594 (1:1000 in 1% BSA, ThermoFisher Scientific, Netherlands) for 1 h at 37 °C followed by PBS washing. Afterward, cells were incubated with mouse anti-goat Alexa Fluor-488 (1:1000 in 1% BSA, Santa Cruz, USA) or anti-rabbit Alexa Fluor-488 (1:1000 in 1% BSA, Invitrogen, USA). Finally, a drop of DAPI (1:1000 in 1% BSA; 1:1000, ThermoFisher Scientific, Netherlands) was added to the hydrogels. The stained samples were removed from the inserts, placed on microscopy slides, and observed under a confocal laser scanning microscope (TCS SP8, Leica, Germany).

#### 2.5.6 Cell Encapsulation

T or S pregelation solutions (0.3 mL) were dispensed in 12-well Thincert cell culture inserts (Greiner Bio-One, GmbH, Kremsmünster, Austria) and conditioned with α-MEM for 90 min (soft gel) at 37 °C and 5% CO_2_. hASCs (1.5 × 10^5^ cells/50 μL) were added inside the soft gel and homogenized by pipetting up and down, and the culture medium (α-MEM) was added on top (0.25 mL) and in the well (1.5 mL). The culture medium was changed every second day. The live/dead assay was performed as described above.

#### 2.5.7 FGF-2 Loading and Bioactivity

The ability of the (glyco)peptide hydrogels to retain and protect growth factors was assessed by injecting FGF-2 (1 μg/mL) in the formed gels. The culture medium (α-MEM) was added on the top and bottom of the hydrogels, and they were maintained at 37 °C and 5% CO_2_. At predetermined time points, the hydrogels were permeabilized with Triton (1%, 3 min), washed with PBS followed by incubation with anti-FGF-2 (1:200, 1 h, room temperature, binds only to bioactive FGF-2), and washed again with PBS. After staining with the secondary antibody (Alexa Fluor 488 anti-mouse, 1:500, 1 h, room temperature) and washing with PBS, the immunostained FGF-2 was imaged using a confocal laser scanning microscope (Leica TCS SP8, Leica Microsystems).

#### 2.5.8 Statistical Analysis

All experiments were performed at least in triplicates. The normality of the data was checked using the Shapiro–Wilk test (*p* < 0.05). Because all data followed a normal distribution, the differences between groups were determined using the *t* test. Statistical significance was defined at different levels (*p* < 0.05, *p* < 0.01, *p* < 0 .001, and *p* < 0.0001). Data are presented as mean ± standard deviation from the independent experiments.

## 3 Results and Discussion

### 3.1 Self-Assembly and Gelation of the Glycopeptide Fmoc-FF-GlcN6S

Fluorenylmethoxycarbonyl diphenylalanine (Fmoc-FF) is the most studied dipeptide used as a building block for supramolecular gels.^11,12,31,32^ Its main advantage is the ability to form gels that are stable at physiological conditions and can be used for a wide range of biomedical applications.^3,11,31,32^ Different experimental conditions, e.g., pH, temperature or solvent switch, are reported to trigger the assembly of Fmoc-FF into long nanofibers that can further organize into an entangled network able to entrap water.^11,32−34^ We glycosylated Fmoc-FF (standard EDC-NHS chemistry, Figure S1) to better mimic the native cellular milieu, the extracellular matrix (ECM), in which most of the proteins are glycosylated. The structure and purity of the obtained glycopeptide, i.e., Fmoc-FF-GlcN6S, were confirmed by ^1^H NMR, MS, FTIR, and HPLC (Figures S2–S4).

The functionalization of Fmoc-FF with GlcN6S can affect the balance of supramolecular forces that drive the assembly of nanofibers and thus alter the gelation ability and/or properties of the formed supramolecular gels, as demonstrated for other glycosylated peptides.^25,26^ We used two methods, namely, temperature (T) or solvent (S) switch (Figure 1B), to trigger the assembly of Fmoc-FF-GlcN6S and studied the effect of the glycosylation by comparison with the nonglycosylated peptide (i.e., Fmoc-FF).

As in the case of Fmoc-FF, the glycopeptide Fmoc-FF-GlcN6S is not soluble in water at room temperature. In the T method, the glycopeptide was suspended in water, and the temperature was raised to 90 °C. Whereas, at these conditions, the peptide amphiphile remained insoluble (Fmoc-FF is soluble at basic pH^11,34,35^), the incorporation of the GlcN6S moiety improved the solubility of the glycopeptide, and we obtained a transparent solution that become viscous upon cooling to room temperature. In the S method, the glycopeptide was dissolved in DMSO, and this solution was then diluted with water to trigger the assembly.^11^ The sol–gel transition of these pregelation solutions was then salt-triggered in different physiologically relevant aqueous solutions, such as buffers and culture media (Figures S6–S10). In this process, the divalent cations from the solution (e.g., Ca^2+^, Mg^2+^) form salt bridges with the negatively charged sulfates of the GlcN6S exposed on the surface of the assemblies (Figure 1B), resulting in gelation (Figure 1C,D).^28,36^ The sol–gel transition for Fmoc-FF-GlcN6S occurred within 30 min for the T pregelation solution and approximately 3 h for the S one, indicating that these methods generated fibers with different properties/abilities to interact with the cations from the media. However, the gels were used or analyzed 24 h after the addition of the buffer to allow the system to reach an equilibrium. Of note, the presence of proteins in the media can also influence the gelation process.^28,37^ Indeed, the rheological measurements demonstrated that protein supplementation of the media increased the stiffness of the gels (Figure S8A,B). Fluorescence spectroscopy (Figure S9) and CD analyses (Figure S10) corroborated the formation of salt bridges and protein binding during the sol–gel transition (details in the SI) and thus the possibility to use the gels as vesicles for protection, encapsulation, and delivery of proteins. Because of these results and the intended biomedical application, in the following studies, we used culture media supplemented with fetal bovine serum (FBS) to trigger the gelation process. At these conditions, we tested three concentrations of the glycopeptide, namely, 5, 7.5, and 10 mM, and observed the formation of gels with an increasing modulus at higher concentrations when using the T method (Figure S7). When applying the S method instead, gelation occurred only at the highest tested concentration (10 mM). We therefore selected the concentration of 10 mM to be used under both methodologies for comparative purposes.

The assembly method can impact significantly the properties of the formed gels by affecting the thickness and mechanical properties of the assembled fibers, the density of cross-linking points between the fibers, and the microstructure, i.e., the distribution of the fibers at a larger length scale.^34^ Atomic force microscopy (AFM, Figure 1C,D) showed the formation of an entangled network of long fibers for both methods. However, a significant difference (*p* < 0.001) between the diameter of the nanofibers assembled by the T method (*d* = 35 ± 7 nm) and the fibers generated by the S method (*d* = 29 ± 4 nm) was observed. This result, together with the different sol–gel transition rate for the S and T method, supports the hypothesis for a different mechanism of fiber growth at the used conditions. Moreover, the density of the cross-linking points between the Fmoc-FF-GlcN6S fibers seems to be higher for the T hydrogels than for the S ones (Figure 1C vs D). These differences are translated in the mechanical properties: gels obtained by the T method had an elastic modulus (*G*′) of 2.4 kPa, whereas the S method resulted in gels with a lower *G*′ of 0.5 kPa (Figure 1E). Fmoc-FF gels prepared by the S method were also assessed for comparative purposes and exhibited *G*′ of 2.1 kPa; i.e., it was significantly higher than the modulus of Fmoc-FF-GlcN6S prepared by the same method. This difference can be due to the formation of longer fibers in the case of Fmoc-FF (challenging to measure because of the entanglement) and/or higher hydration of the glycosylated assembly due to the presence of GlcN6S, showing that not only the preparation method but also glycosylation influenced the properties of the generated gels. We therefore investigated the effect of glycosylation on the molecular organization of the gels by fluorescence spectroscopy, circular dichroism (CD), and Fourier transform infrared (ATR-FTIR) spectroscopy.

**Figure 2:**
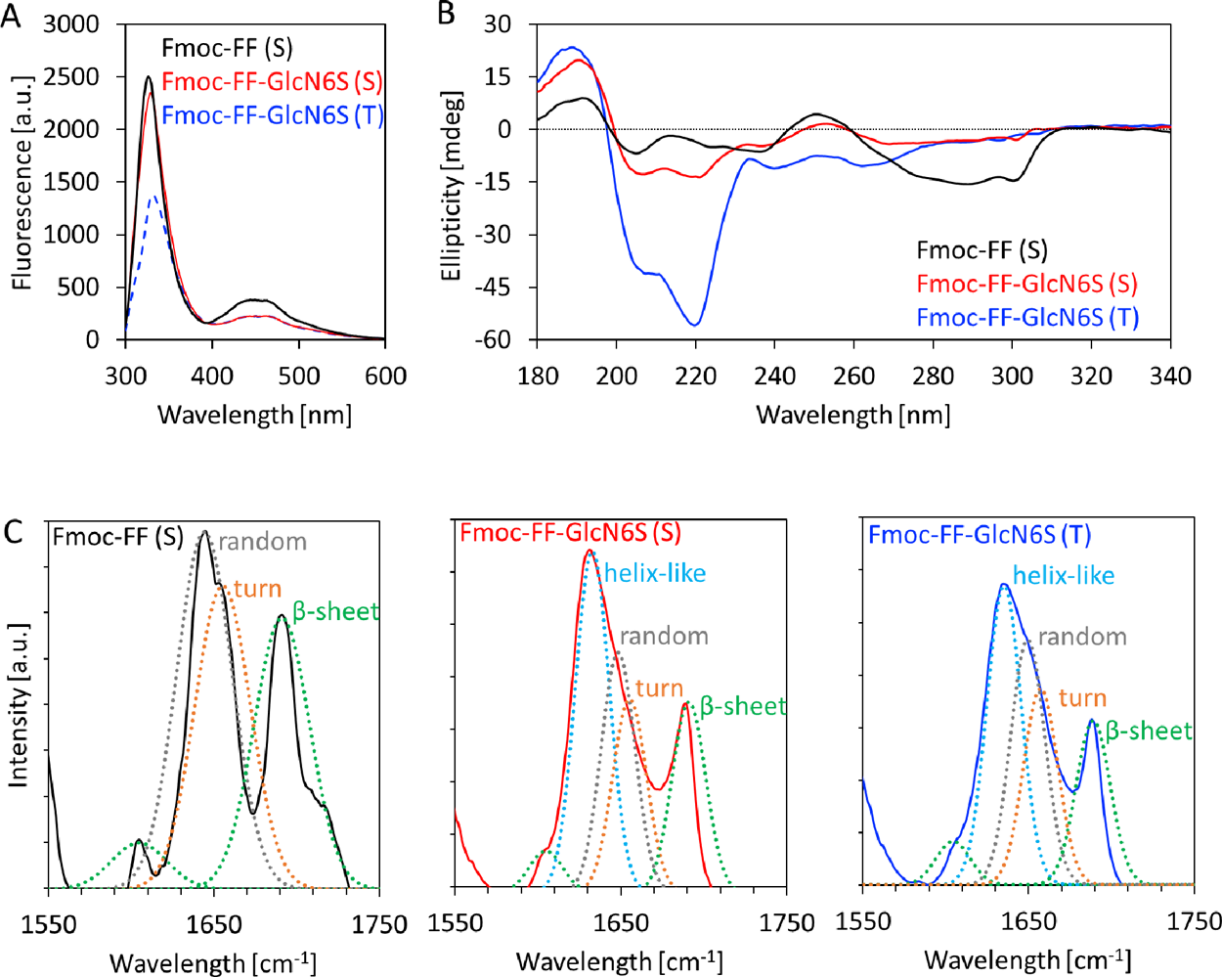
Spectroscopic characterization of molecular organization of the generated gels: (A) fluorescence, (B) circular dichroism (CD), and (C) Fourier transform infrared (ATR-FTIR with the respective Gaussian fitting) spectra of gels assembled from the peptide (Fmoc-FF, 10 mM, S method) and glycopeptide (Fmoc-FF-GlcN6S, 10 mM, S/T methods).

The fluorescence spectra of the Fmoc-FF gel had an intense signal at 328 nm associated with the formation of fluorenyl excimers and a less intense, broad peak at 440–470 nm, consistent with the formation of higher-order aggregates (Figure 2A).^12,32^ In the case of Fmoc-FF-GlcN6S gels, we observed a red shift of the signal at 328 nm (to 333 and 331 nm for the glycopeptide gels obtained by the T or S method, respectively) and a decrease of its intensity (more pronounced for the T gels), evidencing the lower molecular mobility of the fluorenyl moiety in the case of the T gels, i.e., stronger π interactions. However, the intensity of the peak at 440–470 nm is reduced in the spectra of Fmoc-FF-GlcN6S gels when compared to the Fmoc-FF ones (Figure 2A), suggesting that the π–π interactions involving the phenyl and the fluorenyl rings are perturbed in the glycopeptide, most probably because of the formation of CH−π interactions between the introduced carbohydrate moiety and the aromatic groups.^25,38^ Altogether, these data showed a wider intermolecular interactome in the case of Fmoc-FF-GlcN6S whose assembly is driven by H-bonding, CH−π interactions, and π–π stacking, whereas Fmoc-FF does not participate in CH−π interactions.

The CD analysis corroborated these data. The CD spectrum of Fmoc-FF has a positive peak at 194 nm and a negative peak at 206 nm (Figure 2B), corresponding to a β-sheet structure. The spectra of the glycopeptide gels are distinguished by a positive peak at 188 nm and two negative peaks at 208 and 220 nm. These signals indicate the formation of helix-like structures rather than a β-sheet conformation observed for the nonglycosylated peptide.^35^ Whereas an α-helix secondary structure is uncommon in short peptide sequences, helix-like arrangements of different short building blocks have been previously reported.^39^ Of note, the signals for the helix-like structure in the case of Fmoc-FF-GlcN6S were already present in the spectrum of the glycopeptide at higher temperatures (∼90 °C, T method) when fibers are assembled (Figure S6C), and their intensity increased upon temperature drop and over time (Figure S6D), showing conformational stabilization. Moreover, the signal at 260–300 nm is reduced in the Fmoc-FF-GlcN6S spectrum when compared to Fmoc-FF, corroborating the above-mentioned involvement of the fluorenyl moiety in CH−π interactions at the expense of π–π stacking in the case of Fmoc-FF-GlcN6S. Attenuated total reflection Fourier transform infrared (ATR-FTIR) spectroscopy also confirmed the effect of the carbohydrate moiety on the molecular organization. A close look at the amide I region (1600–1800 cm^–1^) and the respective Gaussian fits (Figure 2C) showed an amyloid-like assembly for Fmoc-FF with the typical peaks for β-sheet (1604 and 1691 cm^–1^), random coil (1644 cm^–1^), and turn structures (1655 cm^–1^).^35,40^ In the same region of the ATR-FTIR spectra of the glycopeptide gels (obtained by either the T or S method), a new peak at ∼1635 cm^–1^ emerged consistent with the presence of helix-like supramolecular arrangements.^10,41^

### 3.2 Self-Healing, Biocompatibility, and Biofunctionality of Glycopeptide Gels

**Figure 3:**
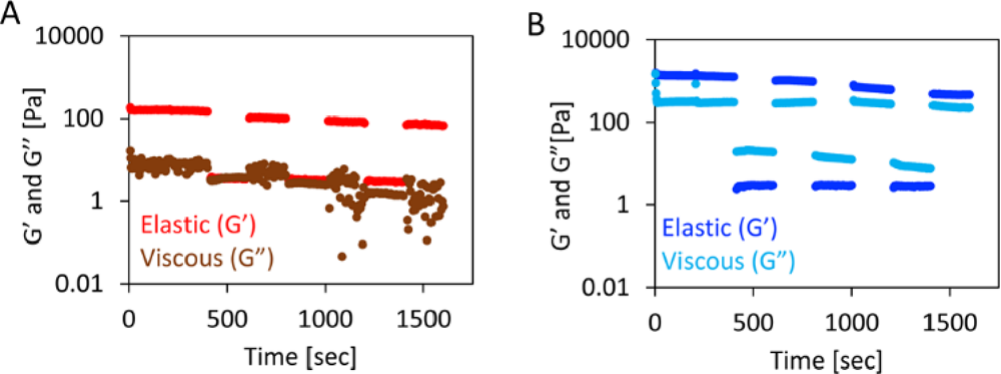
Elastic (*G*′) and viscous (*G*″) components of the shear modulus for glycopeptide gels obtained by (A) solvent (S) and (B) temperature (T) switch methods.

The self-healing capacity of gels is indicative of their ability to recover their shape and mechanical behavior after application of a mechanical stress, e.g., injection. The capacity of Fmoc-FF gels to self-heal has been already described,^42^ and herein, we aimed to check if glycosylation affected this property. We performed a step strain experiment in which gels were subjected to a cycle of sweeps at low and high strain. At the high strain, the hydrogels were converted to a quasi-liquid state (*G*′ < *G*″), and at the low strain, the sol–gel transition occurs (*G*′ > *G*″). We observed self-healing properties for the glycopeptide gels prepared by either of the studied methods (Figure 3). Of note, the gels generated by the S method recovered their shape (stable *G*′ and *G*′ > *G*″) during the whole experiment but presented an unstable *G*″ after two cycles of high strain sweeps. This behavior reflects their lower dissipative ability upon an applied deformation when compared with the gels generated by the T method; i.e., the rheological results confirmed that the gels generated by the T method preserved their self-healing capacity over more cycles (Figure 3B) than the gels obtained by the S method (Figure 3A). These data agree with the observed differences between the gels generated by the T and S method discussed in Section 3.1, i.e., higher entanglement and fiber diameter for T gels.

Direct contact cytotoxicity assays with different cell lines (namely, chondrogenic ATDC5, osteosarcoma SaOS-2, and neuroblastoma SH-SY5Y) showed an excellent biocompatibility of the glycopeptide gels and a significant improvement when compared to the respective peptide gels, in particular at longer culture periods (Figure S12). The striking difference between the peptide and the glycopeptide gels can be explained by the ability of the GlcN6S unit to bind endogenous (expressed by cells) and exogenous (supplemented with the culture media) proteins in a multivalent fashion and preserve their bioactivity, thus mimicking the ECM glycoproteins.^14,19,28^

**Figure 4:**
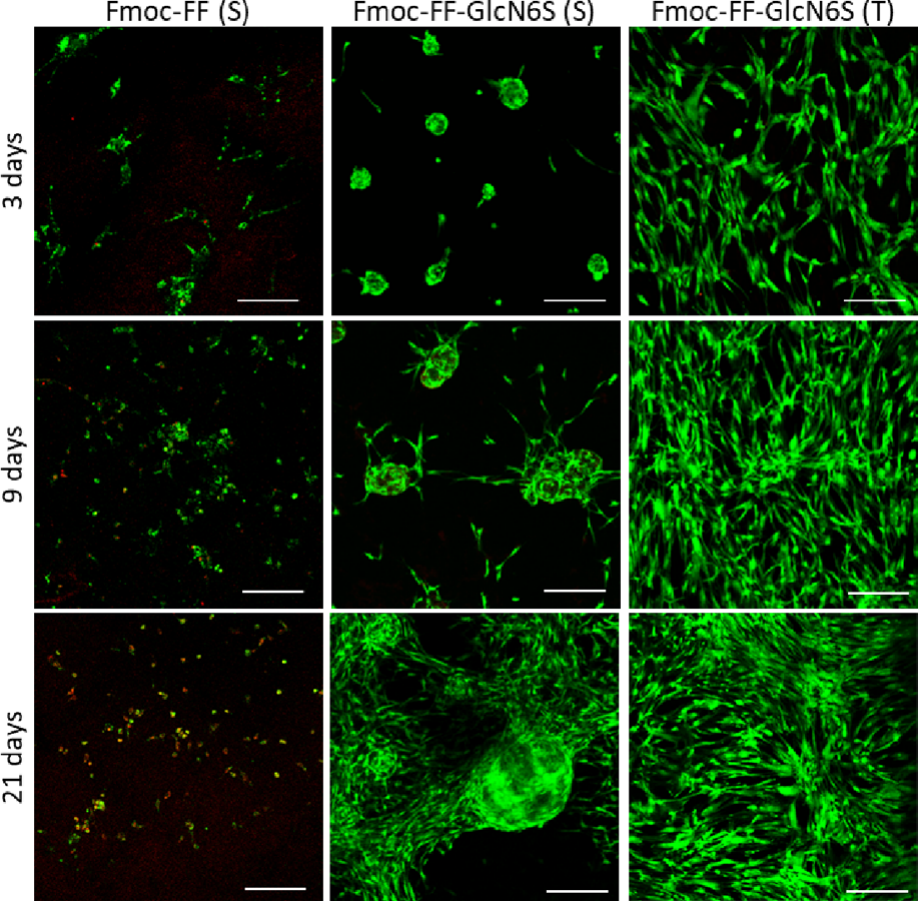
Representative confocal microscopy images of human adipose derived stem cells (hASCs) cultured for different time periods on peptide (Fmoc-FF) and glycopeptide (Fmoc-FF-GlcN6S) gels obtained by solvent (S) or temperature (T) switch methods. Cells were stained with calcein AM (green, live cells) and ethidium homodimer-1 (red, dead cells). Scale bar: 200 μm.

Besides cell lines, the obtained gels were also cytocompatible with primary human adipose stem cells (hASCs, Figure 4). hASCs are multipotent stem cells of the mesenchymal type that can be differentiated *in vitro* into osteoblasts, chondrocytes, adipocytes, myocytes, cardiomyocytes, and neuron-like cells.^43,44^ When compared to other stem cells, hASCs have several advantages: (i) the adipose tissue is abundant and accessible by less invasive procedures, e.g., during aesthetic surgeries; (ii) the isolation is easy, and large quantities can be obtained from a small volume of adipose tissue; and (iii) hASCs have high self-renewal and proliferation capacity.

To induce hASCs’ differentiation, cultures are usually supplemented with soluble factors. As an example, exogenous epidermal growth factor (EGF), basic fibroblast growth factor (FGF-2), retinoic acid, and sonic hedgehog have been used to promote differentiation into neural lineages.^45−47^ The differentiation process is characterized with morphological changes and expression of specific genes, such as Nestin, glial fibrillary acidic protein (GFAP), βIII tubulin, and microtubule-associated protein 2 (MAP2). Fmoc-FF-GlcN6S gels mimic the neural microenvironment in terms of mechanical properties (neural tissue, 0.5–3.0 kPa, Figure S7) and chemical composition (copycatting the sulfated proteoglycans^15,48^), and we hypothesized that these gels can induce neural differentiation without any supplementation with exogenous prodifferentiation stimuli but by capturing, preserving, and activating endogenous growth factors just like the proteoglycans present in the ECM. To confirm this hypothesis, we loaded FGF-2 in the glycopeptide gels and monitored its bioactivity by immunostaining (Figure S15). The results confirmed that the glycopeptide gels preserve the FGF-2 bioactivity throughout 3 days and GlcN6S has a crucial role in this process as no bioactive FGF-2 was observed in the nonglycosylated Fmoc-FF gels (i.e., no staining is observed; Figure S15).

The cytotoxicity assay (Figure 4) evidenced the different behaviors of hASCs cultured on the glycopeptide gels obtained by the T and S methods: we observed more hASCs on the T gels when compared to the S gels. Of note are the morphology and organization of the hASCs: whereas evenly distributed and spread cells with spindle-like morphology were visible on the T gels, on the S gels, cells were organized in spherical aggregates similar to neurospheres that are typically formed by the neuronal stem cells^45,49^ (supplementary videos; Figures S13A, S14, and S16). Such different cell distribution was related with the mechanical properties of the gels: spherical cellular aggregates were also observed on the gels obtained by the T method using a lower concentration of glycopeptide (5 mM, *G*′ ∼0.4 kPa, Figure S16) but not on the gels formed from the pregelation solution at 7.5 mM (*G*′ ∼1.1 kPa, Figure S16) and 10 mM (*G*′ ∼2.4 kPa, Figure S16). Because these gels differ only in their Young’s modulus (same glycopeptide, i.e., Fmoc-FF-GlcN6S, and preparation method, i.e., T, but different concentration), the results suggest that the observed changes in cellular behavior are related with the stiffness of the gels.

**Figure 5:**
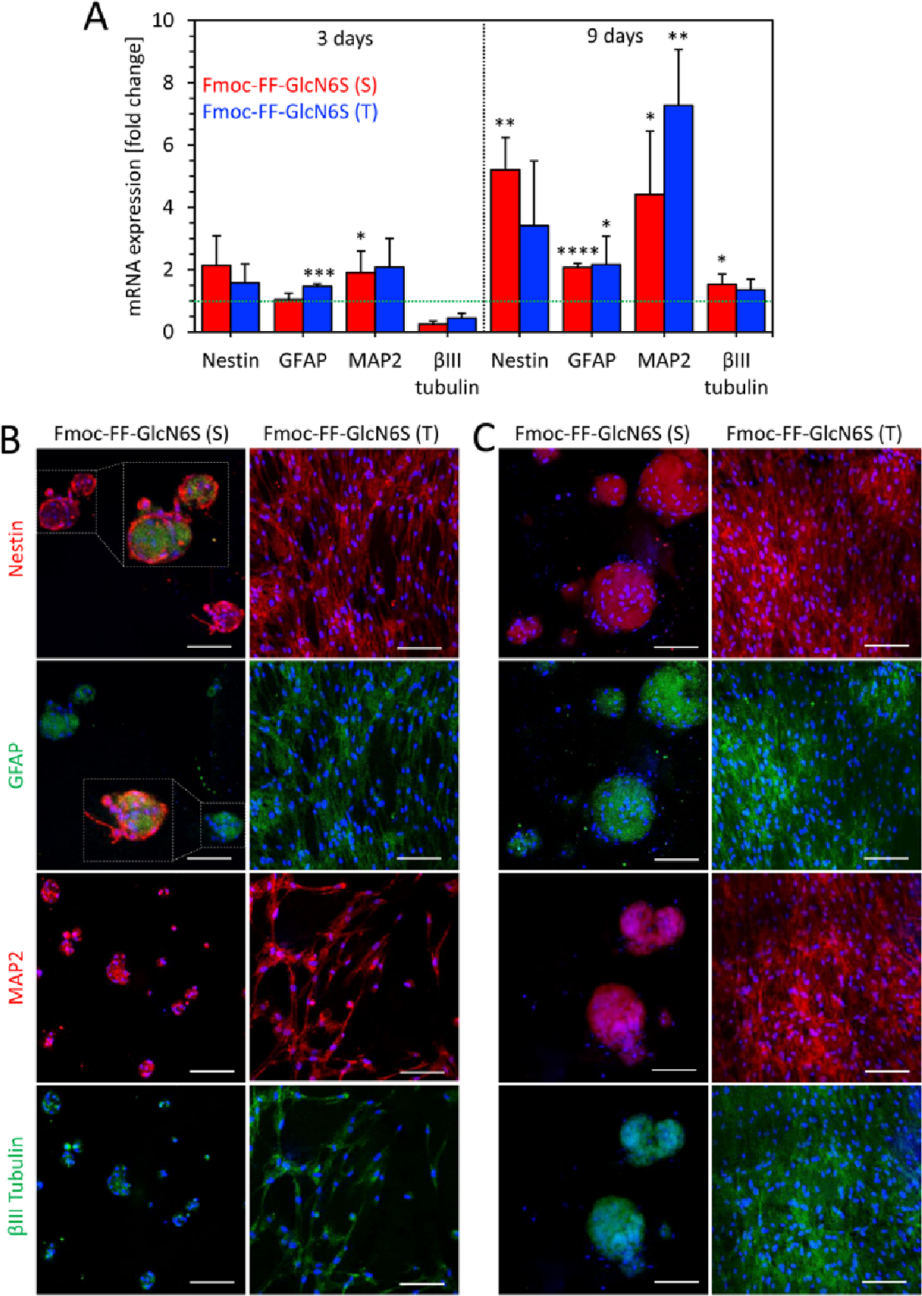
(A) Quantification of the expression of Nestin, GFAP, MAP2, and βIII-tubulin genes by hASCs seeded on glycopeptide gels for 3 and 9 days. Data were normalized to the control (hASCs seeded on tissue culture polystyrene (TCPS), green line). Statistical differences with the control: *** *p* < 0.001, ** *p* < 0.01, and * *p* < 0.05. (B, C) Confocal microscopy images of hASCs seeded on the glycopeptide gels for (B) 3 and (C) 9 days and immunostained for the expression of specific neural proteins: Nestin, GFAP, MAP2, and βIII tubulin. Nuclei were stained in blue. Scale bar: 100 μm. Controls (hASCs on TCPS and peptide gels) are shown in the SI (Figure S17) and do not express any of the studied proteins.

We quantified the expression of the specific genes associated with the neural differentiation (Figure 5A). In general, we observed an upregulation of the neural and glial genes already at day 3 (namely, MAP2 and GFAP) as well as at day 9 (Nestin, GFAP, MAP2, and βIII tubulin). The upregulation was more pronounced and statistically significant when compared to the control (tissue culture polystyrene (TCPS)). Importantly, at this time point, βIII tubulin was also upregulated. The immunostaining (Figure 5B,C) corroborated the PCR analysis: we observed an overexpression of all tested proteins when compared to the controls (i.e., Fmoc-FF gels and TCPS; Figure S17), in particular, Nestin and GFAP, which suggests the presence of neural stem and progenitor cells, whereas the expression of MAP2 and βIII tubulin confirms the development of neural cell phenotypes. In the case of hASCs cultured on S gels, we observed cellular organization that is specific for neurospheres with Nestin-positive cells on the surface of the spheroid and GFAP-positive cells in the core^49^ after 3 days of culture. In contrast, the surface of the T gels was homogeneously populated with Nestin-positive cells (Figure 5B). In addition, hASCs cultured on S gels expressed more Nestin and βIII tubulin when compared to the cells cultured on T gels. These results further support the hypothesis that, in addition to biochemical signals (i.e., glycosylation), the adjustable stiffness of the glycopeptide gels can be used to tune cellular response.

**Figure 6:**
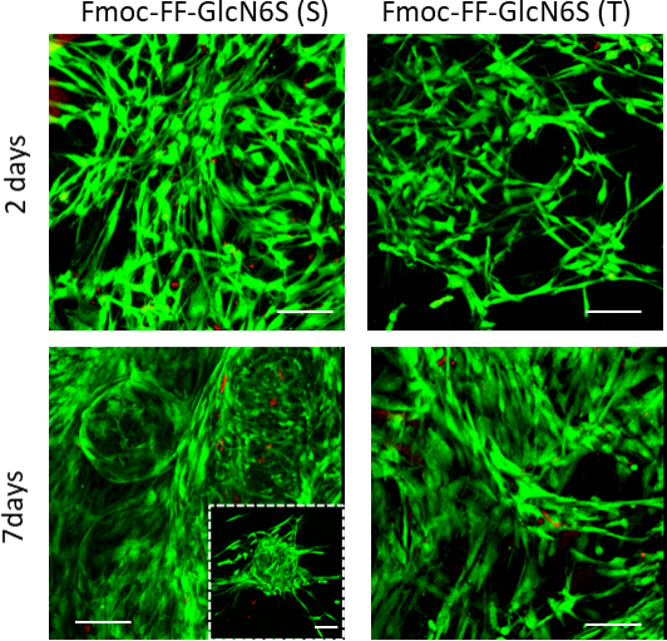
Confocal microscopy images of hASCs encapsulated in the glycopeptide gels for 2 and 7 days and stained with calcein AM (green, live cells) and ethidium homodimer-1 (red, dead cells). Scale bars: 200 μm; 50 μm for the inset image.

The application of stem cells in regenerative therapies requires the encapsulation of cells *in vitro* followed by their delivery *in vivo*. However, the temperature cycle necessary to generate the Fmoc-FF-GlcN6S nanofibers may compromise the encapsulation process and the use of the gels as an injectable system. We therefore tested the possibility to encapsulate hASCs in the S/T glycopeptide gels. We prepared a viscous glycopeptide pregelation solution by heating–cooling in the case of the T method, and at this step (i.e., prior the salt-triggered gelation), hASCs were added. In the case of the S-based gels, we used them at the initial steps of the gelation stage (as previously mentioned, it takes approximately 3 h to form a stable gel), and the cell suspension was mixed with the gel. In both cases, the cross-linking was initiated and allowed to proceed by adding culture media. This procedure resulted in the encapsulation of viable cells whose morphology and organization were similar to the ones observed under 2D culture (Figure 6). These results demonstrate that the developed glycopeptide gels are a versatile biofunctional tool that can be used in different approaches for the regeneration of neural tissues, e.g., for the treatment of spinal cord injuries.

## 4 Conclusions

We obtained biofunctional supramolecular hydrogels from a minimalistic glycopeptide. The obtained gels performed significantly better than the respective analogue generated using the nonglycosylated peptide in terms of (1) cytocompatibility; (2) capturing and preserving growth factors (e.g., FGF-2); (3) maintenance of cell cultures for longer periods (>3 days); and (4) stem cell differentiation into neural lineages. These advantages are due to the synergistic integration of several properties into the developed ECM mimicking gels, namely, hydrated structure, nanofibrillar network, appropriate stiffness, and biofunctional chemistry that copycat the interactions of negatively charge proteoglycans from the ECM with endogenous signaling proteins. The versatility of the proposed glycopeptide system was demonstrated by the use of different methodologies for the formation of the gels, whereas the applicability was evidenced by differentiation of hASCs into neural lineages.

## Supporting Information Available

Additional experimental results (PDF)
Video tracking the adhesion and spreading of live cells cultured on the glycopeptide hydrogel (obtained by the T method) during 11 days (MPG)
Video tracking the adhesion and spreading of live cells cultured on the glycopeptide hydrogel (obtained by the S method) during 11 days (MPG)
Video tracking the adhesion and spreading of live cells cultured on peptide hydrogel (obtained by the S method) during 11 days (MPG)
Video tracking the adhesion and spreading of live cells cultured on TCPS during 11 days (MPG)

## Supplementary Material

am3c05309_si_001.pdf

am3c05309_si_002.mpg

am3c05309_si_003.mpg

am3c05309_si_004.mpg

am3c05309_si_005.mpg
