# Supplementary material for: Glycopeptide-Based Supramolecular Hydrogels Induce Differentiation of Adipose Stem Cells into Neural Lineages
Source: ACS Appl Mater Interfaces. 2023 Jun 16;15(25):29998–30007. doi: 10.1021/acsami.3c05309 (PMC10316323; doi:10.1021/acsami.3c05309)
Supplement: Supplementary file 1 — am3c05309_si_001.pdf [file am3c05309_si_001.pdf]

SUPPORTING INFORMATION

Glycopeptide-Based Supramolecular Hydrogels Induce Differentiation of Adipose Stem Cells into Neural Lineages

Vânia I. B. Castro,[a,b] *Ana R. Araújo,*[a,b] *Filipa Duarte,*[a,b] *António Sousa-Franco*[a,b]*, Rui L. Reis,*[a,b] *Iva Pashkuleva,*[a,b*] *Ricardo A. Pires*[a,b*]

[a] 3B's Research Group, I3Bs – Research Institute on Biomaterials, Biodegradables and Biomimetics, University of Minho, Headquarters of the European Institute of Excellence on Tissue Engineering and Regenerative Medicine, 4805-017 Barco, Portugal

[b] ICVS/3B's–PT Government Associate Laboratory, 4800 Braga/Guimarães, Portugal

[*] E-mail: pashkuleva@i3bs.uminho.pt; rpires@i3bs.uminho.pt

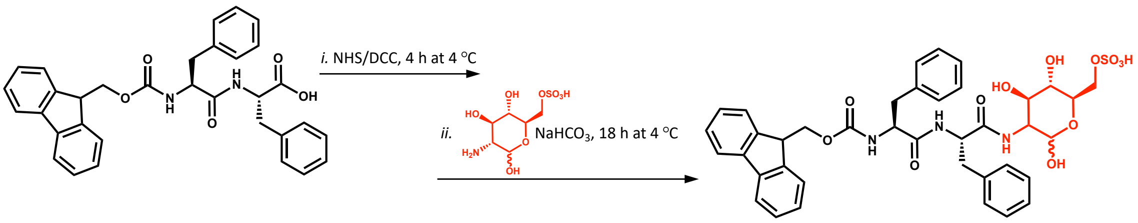

Figure S1. Schematic presentation of the synthetic procedure used to obtain the glycopeptide Fmoc-FF-GlcN6S.

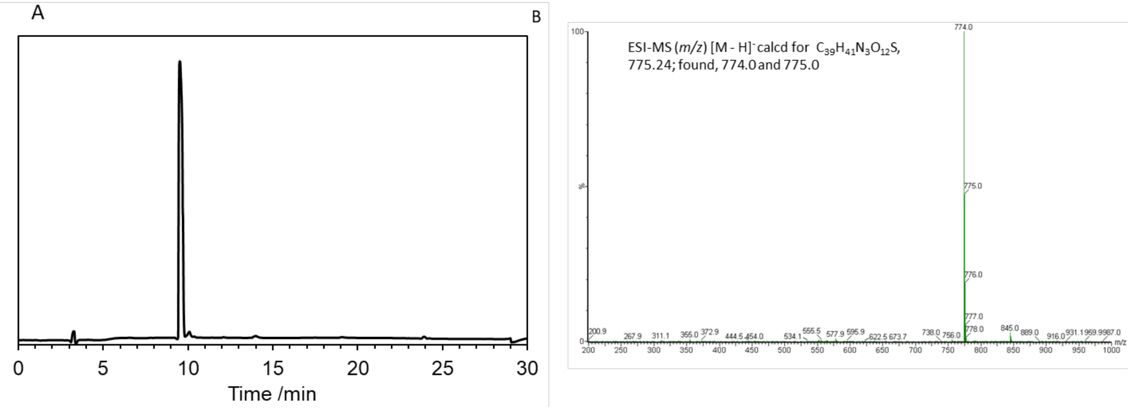

Figure S2. (A) HPLC chromatogram of the purified glycopeptide Fmoc-FF-GlcN6S; (B) ESI-MS spectrum (negative-ion mode) of the glycopeptide Fmoc-FF-GlcN6S $[M-H]^-$: m/z= 774.0; $M(C_{39}H_{41}N_3O_{12}S)$ = 775.24g/mol.

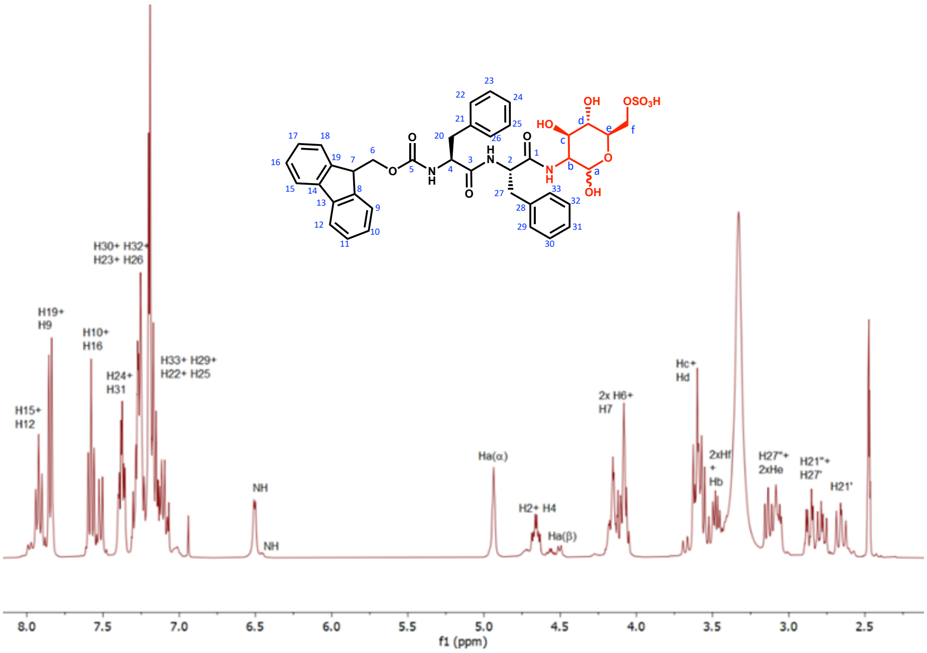

Figure S3. ^{1}H NMR spectrum (400MHz, DMSO-d_6) of the glycopeptide Fmoc-FF-GlcN6S.

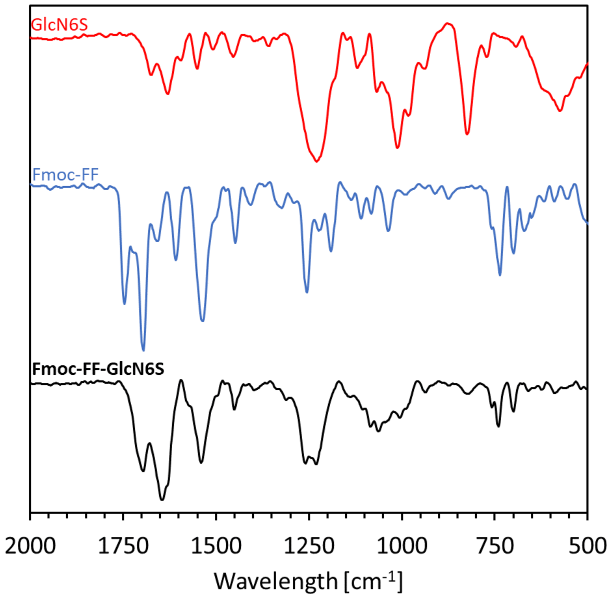

Figure S4. FTIR spectra of glucosamine-6-sulphate (GlcN6S, red), the peptide amphiphile Fmoc-FF (blue) and the glycopeptide amphiphile Fmoc-FF-GlcN6S (black).

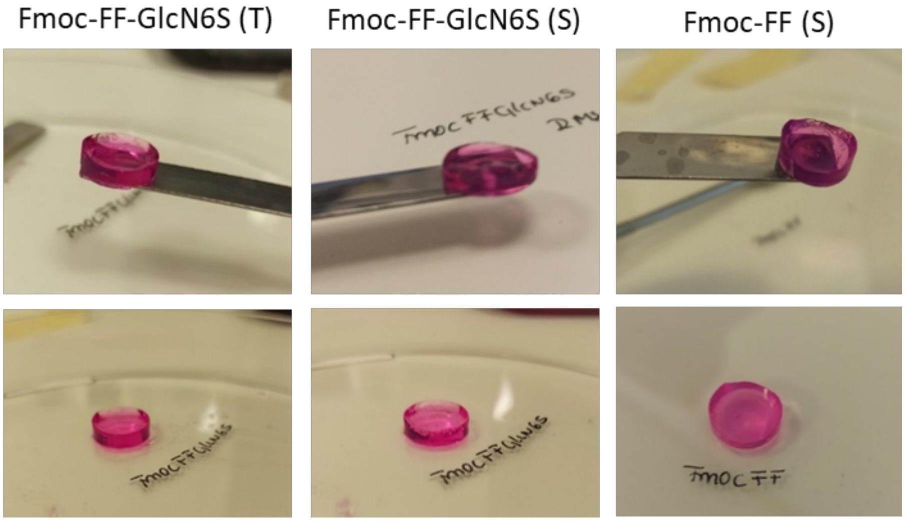

Figure S5. Macroscopic appearance of the gels obtained from pre-gelation solutions (10mM) using the peptide (Fmoc-FF) or glycopeptide (Fmoc-FF-GlcN6S) in contact with culture medium (α-MEM).

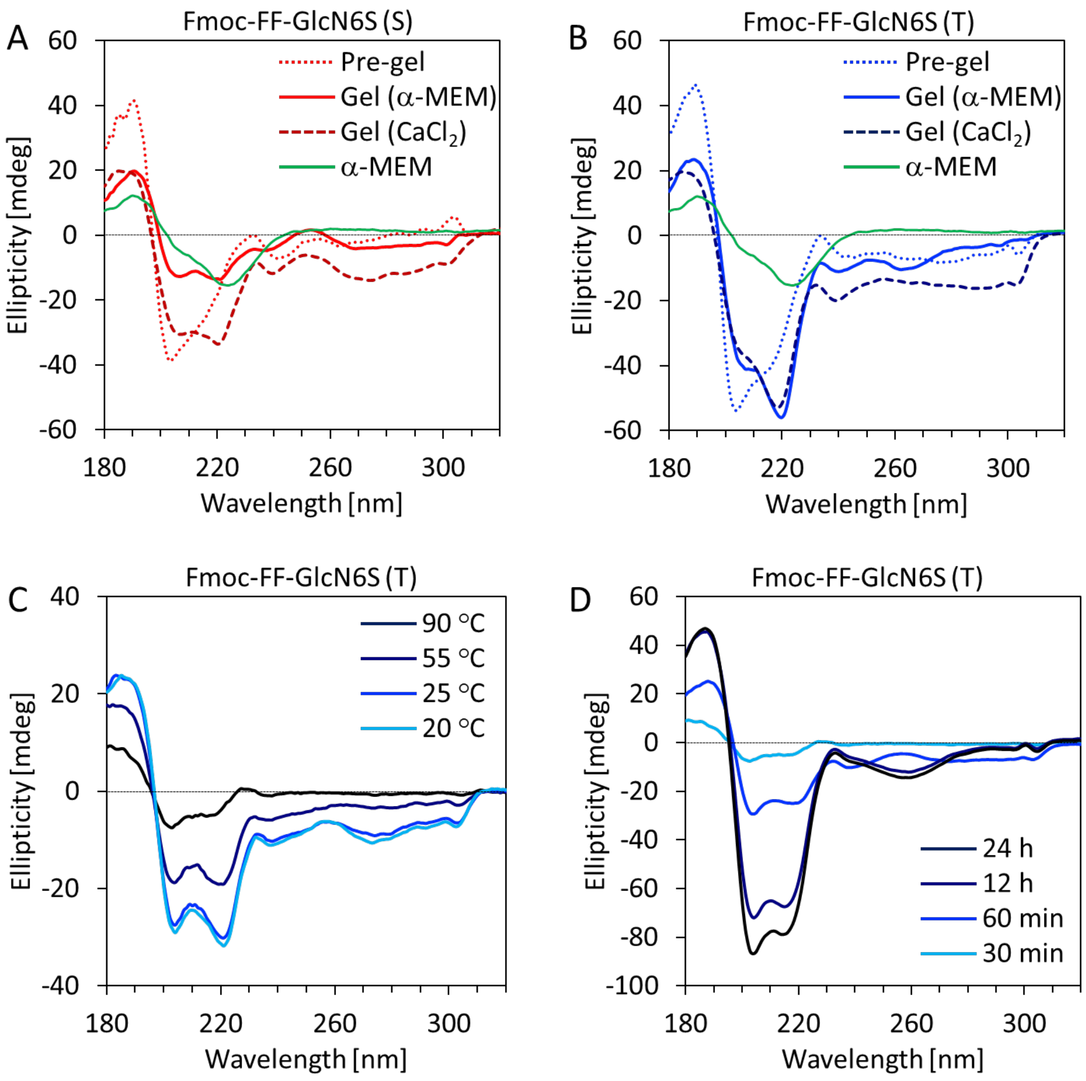

Figure S6. CD spectra of the pre-gelation solutions (10mM) and the respective gels of the glycopeptide (Fmoc-FF-GlcN6S) generated upon contact with different media obtained by (A) S method and (B) T method. (C) Induction of self-organization upon temperature decrease (T method). (D) Stability of the assemblies prepared by the T method as a function of time.

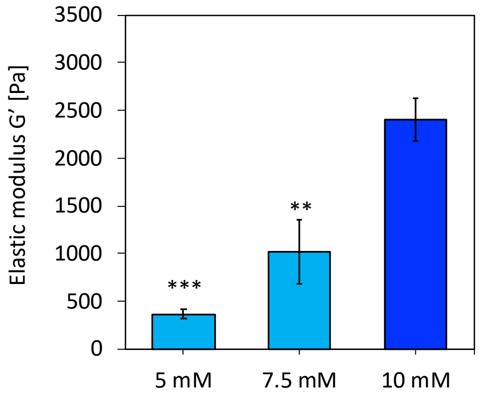

Figure S7. Elastic modulus of glycopeptide gels obtained by the T method from pre-gelation solutions with different concentrations. Gelation was induced in α-MEM at 37°C for 24h. Statistical significance to 10 mM gel: *** $p < 0.001$, ** $p < 0.01$.

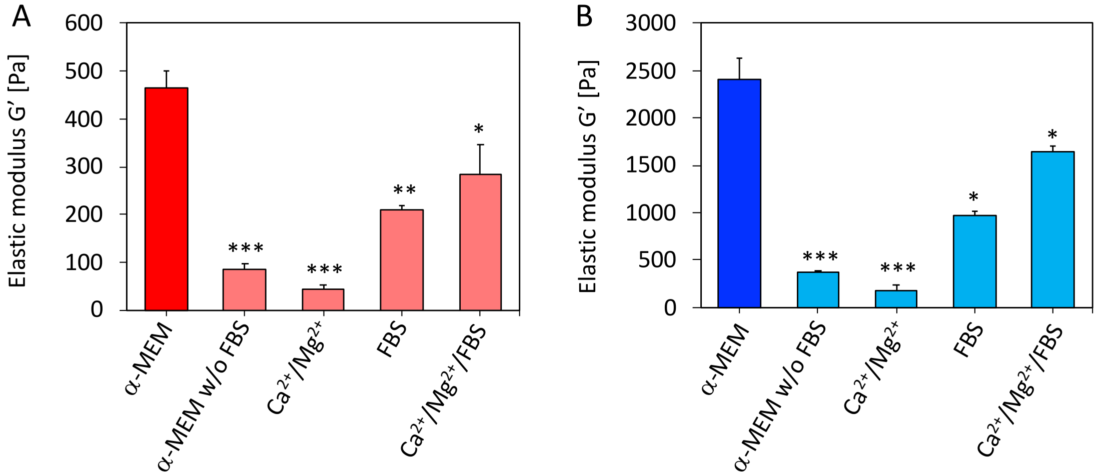

Figure S8. Elastic modulus (G') of the glycopeptide gels (10mM) prepared by (A) S method or (B) T method and different media to induce gelation at 37°C. Statistical significance to the gel obtained in α-MEM: *** $p < 0.001$, ** $p < 0.01$, * $p < 0.05$.

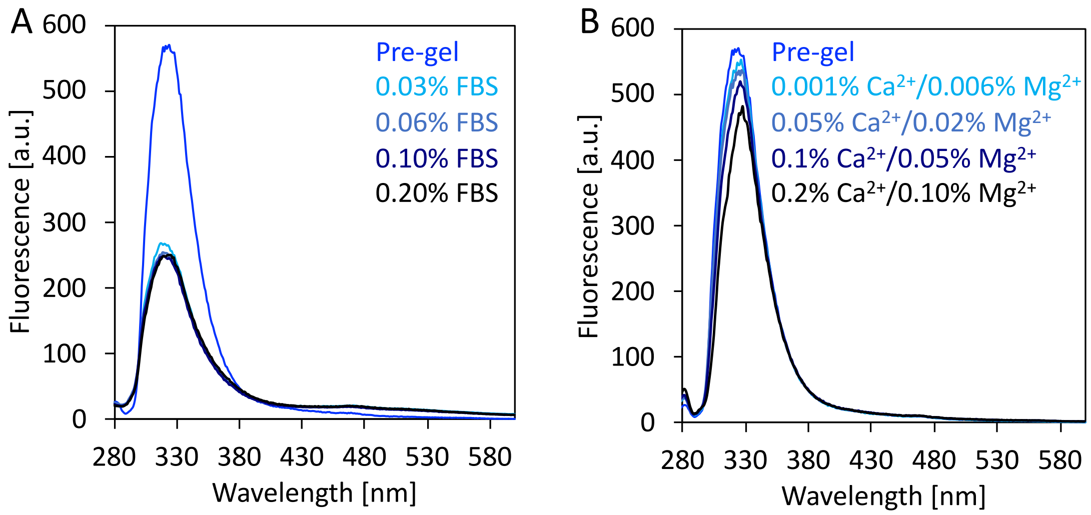

Figure S9. Fluorescence spectra of the glycopeptide pre-gelation solution (10mM, T method) and (A) gels generated in media with fetal bovine serum (FBS) at different concentrations or (B) gels generated in media with different concentrations of bivalent cations.

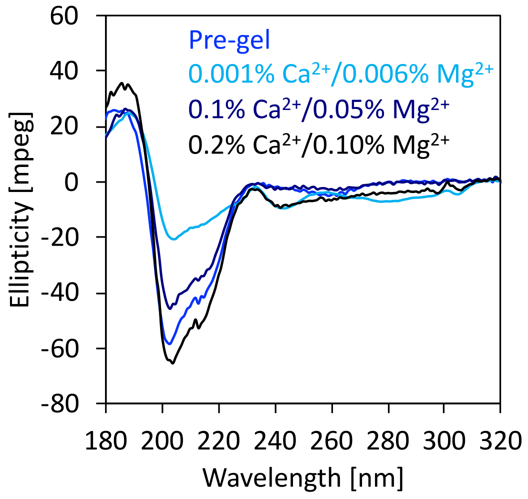

Figure S10. CD spectra of the glycopeptide pre-gelation solution (10mM) prepared by the T method and exposed to media with different concentration of bivalent cations Ca^{2+} and Mg^{2+}.

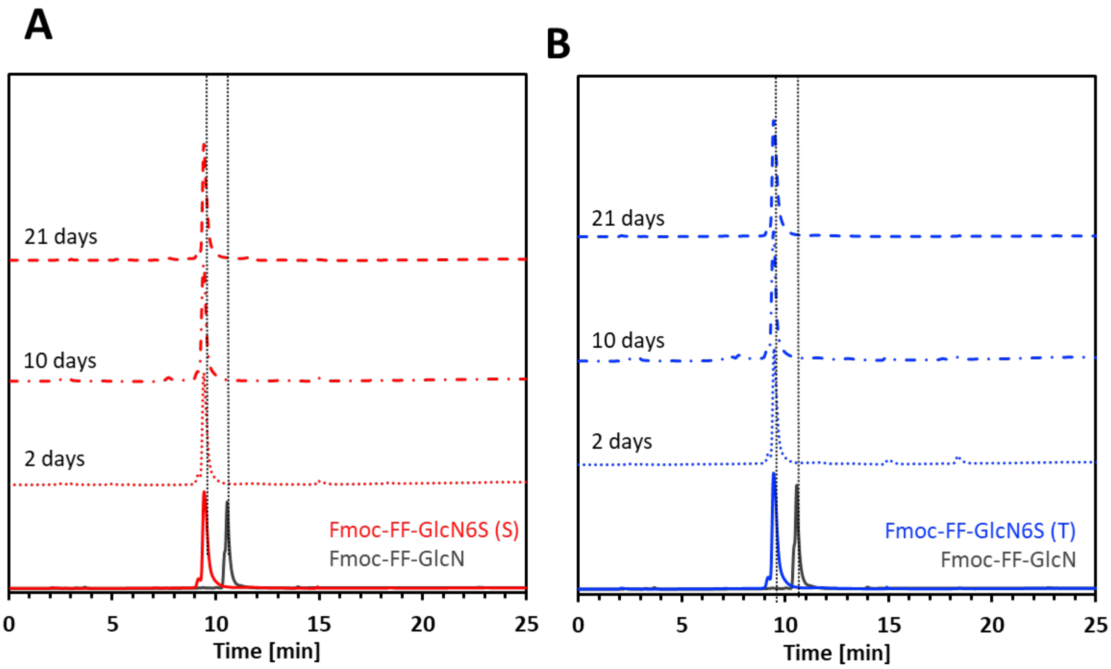

Figure S11. HPLC chromatograms of the glycopeptide (Fmoc-FF-GlcN6S) gels obtained by (A) S method (red lines) and (B) T method (blue lines) in α-MEM at 37°C and 5% CO_2 for 2, 10 and 21 days. Grey line corresponds to the Fmoc-FF-GlcN (glycopeptide without 6-sulfate) used as a control sample.

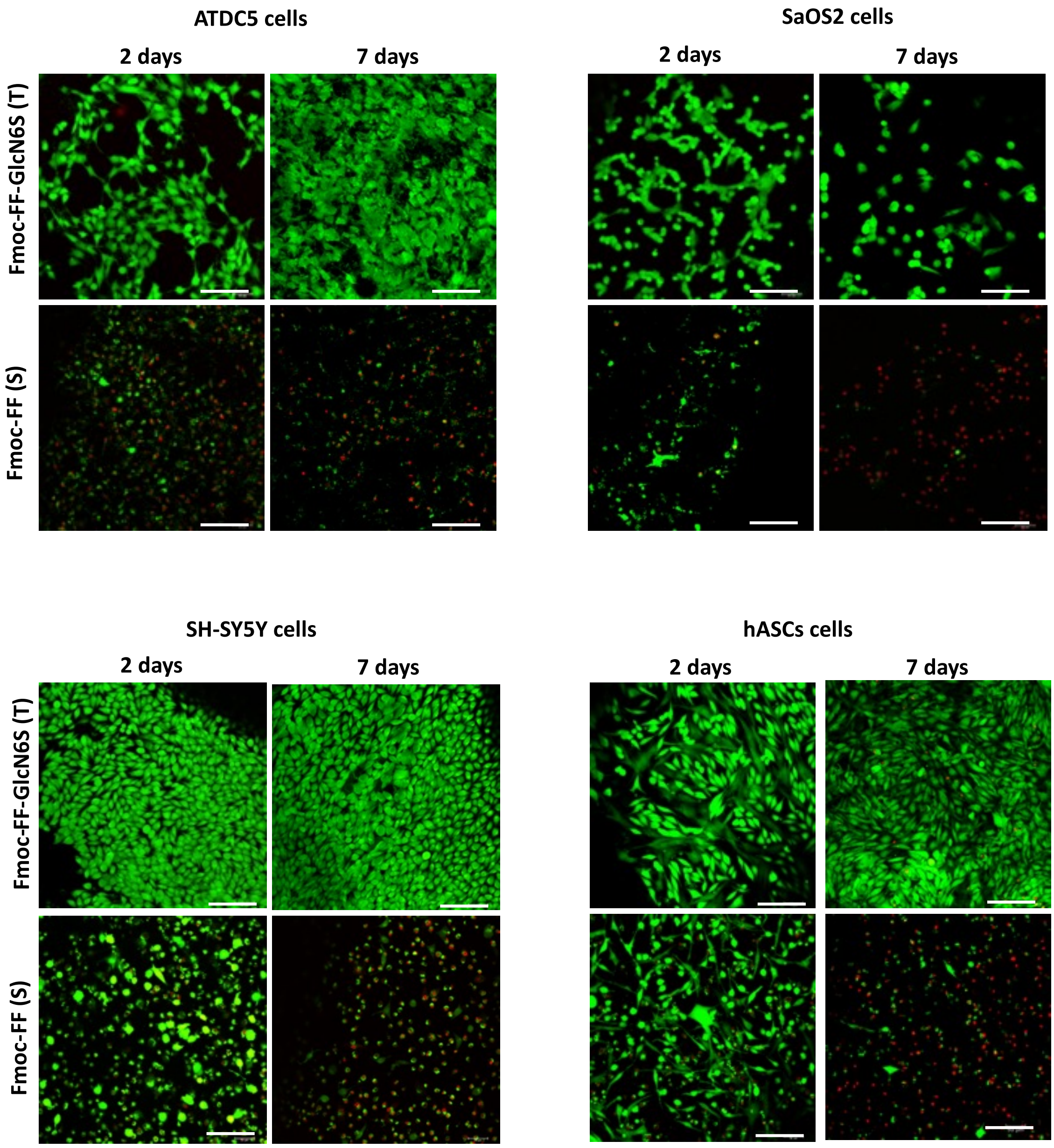

Figure S12. Confocal microscopy images of cells seeded on peptide (Fmoc-FF) and glycopeptide (Fmoc-FF-GlcN6S) gels (10mM, T method) and stained with calcein AM (green, live cells) and ethidium homodimer-1 (red, dead cells). Scale bar: 200 µm.

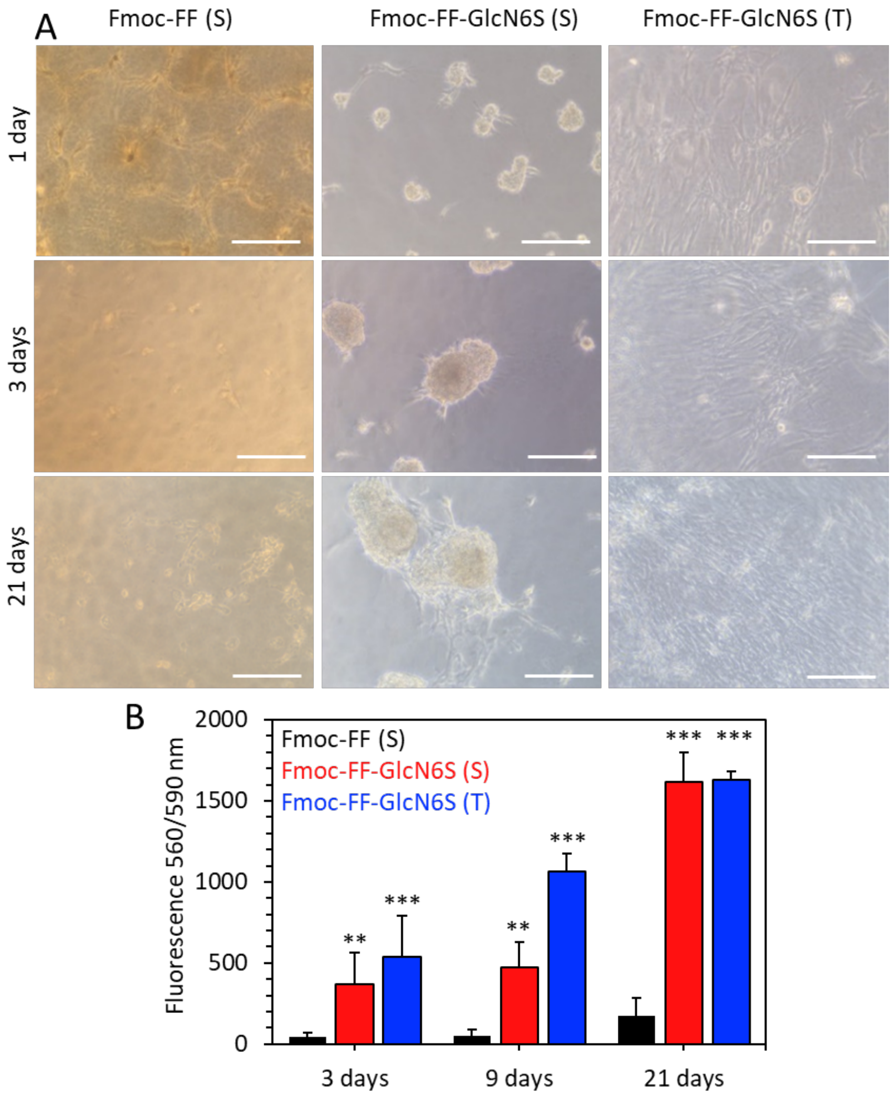

Figure S13. (A) Phase-contrast microscopy images and (B) metabolic activity (AlamarBlue® assay) of hASC cultured on the peptide and glycopeptide gels (10mM in α-MEM) for different timeframes. Scale bar 200 μm. Statistical difference to Fmoc-FF gels: *** $p < 0.001$, ** $p < 0.01$.

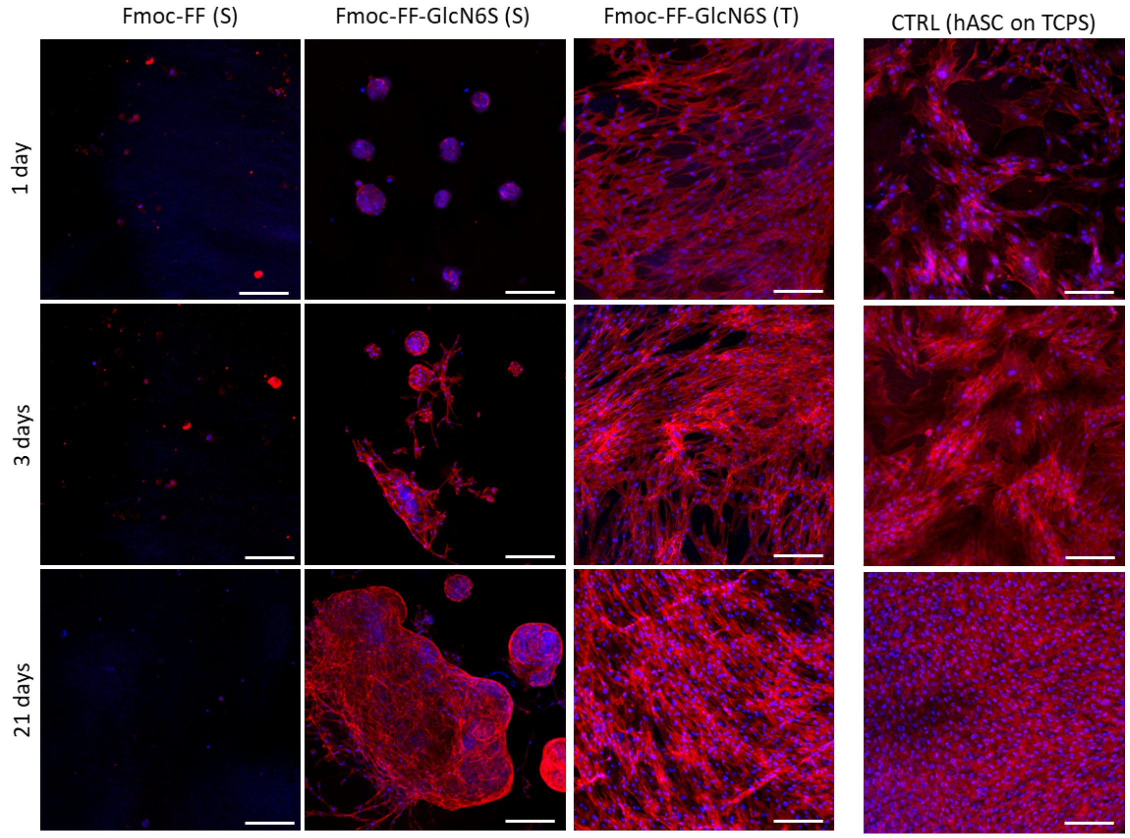

Figure S14. Confocal microscopy images of hASC seeded on peptide (Fmoc-FF) and glycopeptide (Fmoc-FF-GlcN6S) gels (10mM in α-MEM) for 3, 9 and 21 days and stained with DAPI (blue, cell nuclei) and phalloidin (red, cytoskeleton). Scale bar: 200μm.

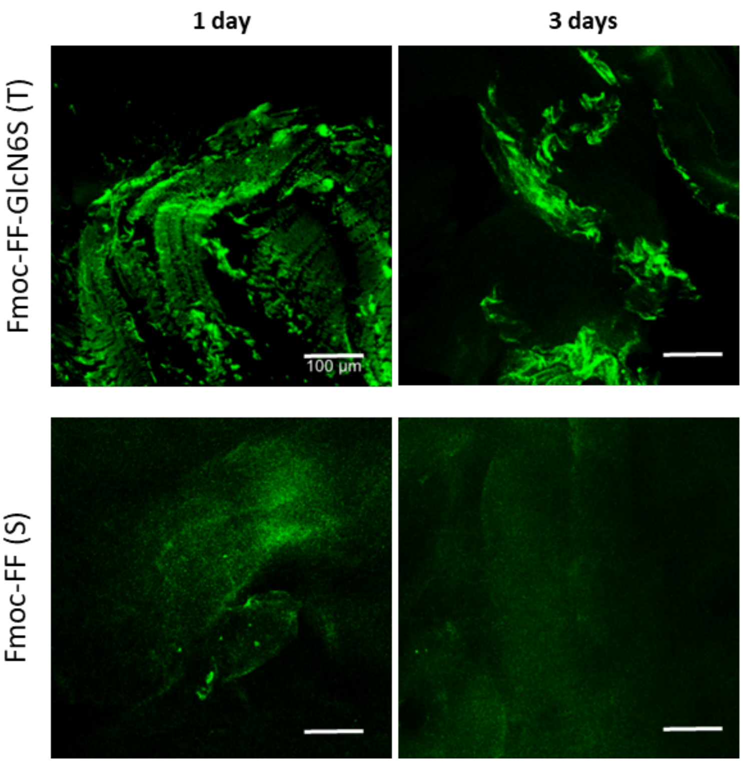

Figure S15. Confocal microscopy images showing FGF-2 distribution and stability within the glycopeptide (Fmoc-FF-GlcN6S) and the peptide (Fmoc-FF) gels. FGF-2 was immunostained with anti-FGF-2 (binding only to active conformation of FGF-2) and a secondary Alexa Fluor 488 antibody. Fmoc-FF gels were generated by the S method and Fmoc-FF-GlcN6S gels were obtained by the T method.

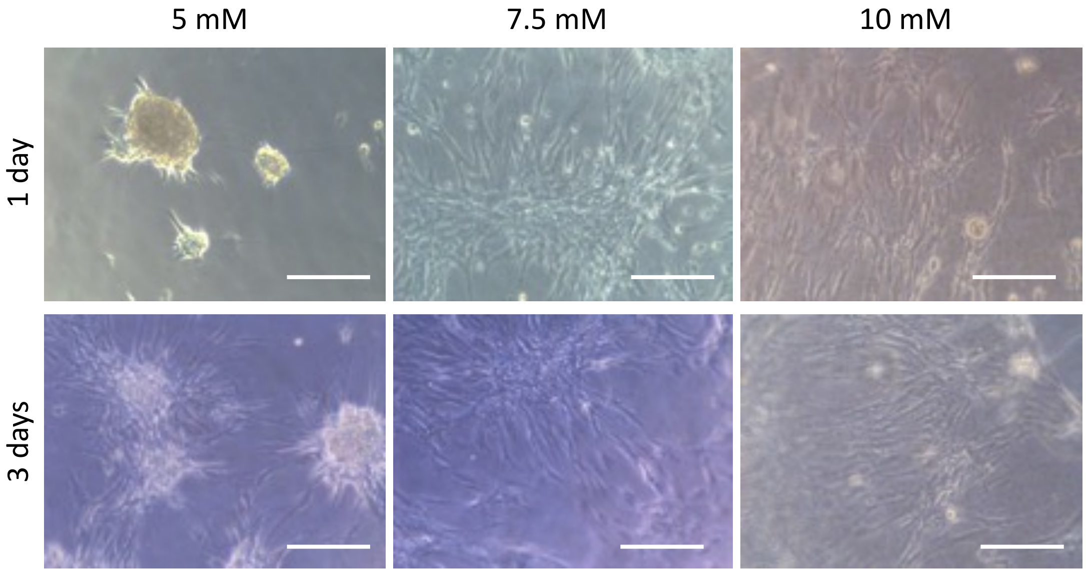

Figure S16. Effect of glycopeptide (Fmoc-FF-GlcN6S) concentration (and mechanical properties) of the T gels on the cellular organization. Scale bar: 200μm.

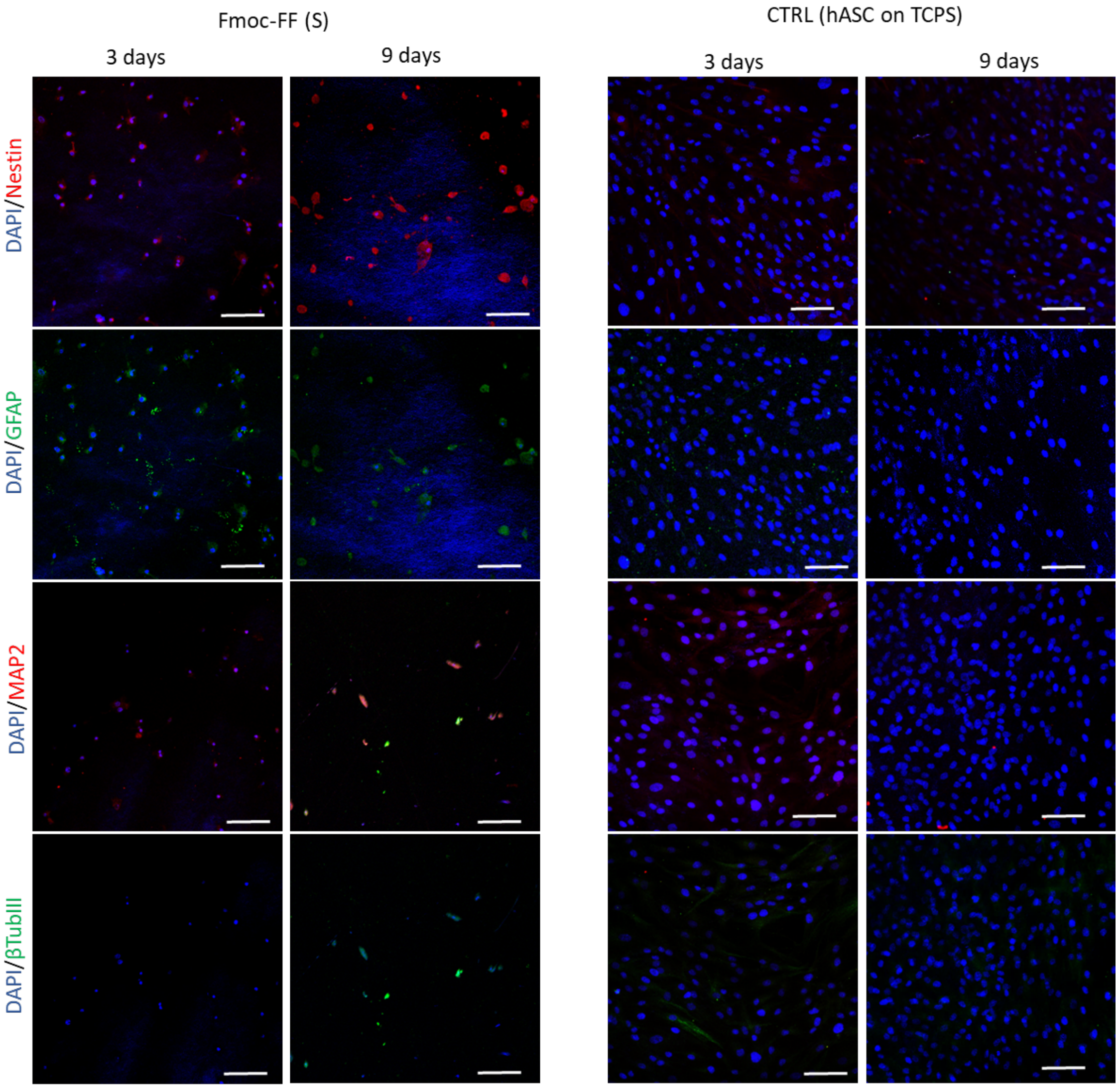

Figure S17. Confocal microscopy images of immunostained hASC seeded on control samples, i.e., peptide (Fmoc-FF) and TCPS, at 3 and 9 days of culture. Scale bar: 100μm.

Table S1. Real-time PCR primers used for quantifying the mRNA expression

Gene	Primer	
Nestin	forward	AACAGCGACGGAGGTCTCTA
	reverse	TTCTCTTGTCCCGCAGACTT
GFAP	forward	GAGTCCCTGGAGAGGCAGAT
	reverse	GTAGGTGGCGATCTCGATGT
bIII tubulin	forward	ACCTCAACCACCTGGTATCG
	reverse	TTCTTGGCATCGAACATCTG
MAP2	forward	GGCATTGAAGAATGGCAGAT
	reverse	TCTCCGTTGATCCCATTCTC

Videos 1-4. Time lapse images showing the adhesion and spreading of hASC on:

Video 1 - Glycopeptide (Fmoc-FF-GlcN6S) (10mM) gel obtained by the T method.

Video 2 - Glycopeptide (Fmoc-FF-GlcN6S) (10mM) gel obtained by the S method.

Video 3 - Peptide (Fmoc-FF) (10mM) gel obtained by the S method.

Video 4 - Control substrate (tissue culture polystyrene, TCPS).
